# Bridging the gap to clinical practice: a concept for virtual patients in preclinical education in prosthetic dentistry

**DOI:** 10.1186/s12909-025-08097-4

**Published:** 2025-10-16

**Authors:** Marco S. Schulz, Ragna Pantelmann, Anna-Lena Hillebrecht, Daniel R. Reissmann

**Affiliations:** 1 Praxis Dr. Schlendermann, Lüneburg, Germany; 2 Department of Prosthetic Dentistry, Center for Dental and Oral Medicine, University Medical Center Hamburg-Eppendorf, Hamburg, Germany; 3 Department of Prosthetic Dentistry, Center for Dental and Oral Medicine, University of Freiburg, Freiburg, Germany; 4 Department of Prosthodontics and Materials Science, University of Leipzig, Liebigstr. 12, Leipzig, 04103 Germany

**Keywords:** Dental education, Digitization, Distance learning, E-learning, Virtual patients

## Abstract

**Background:**

As the transition to clinical practice is accompanied by challenges, the aim of this study was to develop and evaluate a concept for the integration of virtual patients in the dental curriculum at a dental school in Germany.

**Methods:**

The defined learning goals were converted into corresponding modules, which consist of three main components: Case information, additional information and learning tasks. The modules served as a guideline for converting the existing case documentation into a coherent case and were supplemented with additional information. Learning tasks were integrated under the aspect of case-based learning. At baseline, 48 students participated (response rate: 90%) while the follow-up was completed by 36 students (response rate: 68%), of whom 24 stated to have used the e-learning tool.

**Results:**

Statistically significant improvements in perceived competence for users were found in almost every item, and the overall agreement increased from 8.3% to 75%. For participants who did not use the e-learning tool, the overall agreement only increased to 33.3% with only few statistically significant improvements. In the evaluation of the e-learning tool, an overall agreement of 75% was documented regarding functionality and satisfaction. The scope reached an overall agreement of 87.5%.

**Conclusion:**

The results show a high level of student’s satisfaction and improvements in terms of perceived competence, achieved by the concept-based integration of virtual patients. It can therefore be seen as a promising incorporation into the existing curriculum.

**Supplementary Information:**

The online version contains supplementary material available at 10.1186/s12909-025-08097-4.

## Introduction

Dental treatment is all about patients. However, exposure to real patients first occurs in clinical study section, while training is initially conducted on phantom patients, where standardized cases are treated according to defined procedures. General anamnestic and social backgrounds do not play a role at this stage, although interdisciplinarity is becoming increasingly important. This may not adequately prepare students for the high demands they will face in the care and treatment of real patients [1, 2]. Individual dental and medical histories add complexity, which may lead to novice clinical students feeling strongly challenged or even overwhelmed [3]. Soft skills (e.g., empathy, active listening, effective communication) are crucial for competent patient care, but yet are only sparsely and inconsistently addressed in dental curricula [4, 5]. For this reason, transition from preclinical education to clinical practice is challenging, and contact with patients should therefore be facilitated at an earlier stage.

Following a constructivist learning approach, a curriculum should aim to create rich, multimodal, and communication-oriented environments that address subjective experiential domains while simultaneously incorporating new “puzzles” that pragmatically, interactively and creatively invite self-orientation [6]. In traditional classroom teaching, students` active participation is limited to a certain level and the lecturer remains as the center of the teaching process [7]. Students primarily act as passive listeners, to whom knowledge is imparted in a monotonous and unattractive way [8]. This approach lacks enthusiasm and inspiration and does not effectively address the challenges mentioned above. E-learning prioritizes students and provides them with a secure and consistent learning environment [6, 9]. Students can progress according to their individual prerequisites and needs without being constrained by location or time. Developing a concept for utilizing virtual patients (VPs) is one potential strategy for the incorporation of e-learning in the dental curriculum. Patient scenarios can vary in complexity, benefiting both, novice as well as advanced students [3]. They can explore topics of their own interest, and self-directed progress leads to a more personalized learning experience [10]. Within the e-learning environment, they can manage long-term cases in a shorter timeframe and gain initial patient experience without exposing actual patients to any risk [11]. Therefore, the integration of virtual patients in dental education becomes increasingly important nowadays, as they help to overcome limited clinical exposure, address increasing curricular demands, and promote digital skills that are essential for future healthcare professionals.

There is great interest in development and use of VPs in medical education in general as well as in the dental curriculum in particular [12, 13]. They are a helpful tool and may play an increasingly important role in future education [3]. A study on the use of VPs in urology showed a better learning outcome in comparison to a textbook group [11]. Students’ interest in urology was increased, and they would recommend it to fellow students. VPs are used in a variety of ways in dentistry, too, and have proven to be a useful addition to the curriculum [14]. For example, undergraduate dental students had a positive attitude in regard of VP cases in oral surgery. In combination with traditional teaching a significant increase of knowledge in comparison to traditional teaching alone was reported [15]. In an oral and maxillofacial surgery clerkship, dental students found VPs to be a “worthwhile learning experience” and a promising substitute to lecture-based, small-group teaching [16]. Furthermore, the use of VPs in the context of herpes simplex virus infection and recurrent aphthous stomatitis demonstrated improved learning and clinical decision making [17]. In addition, Goodacre highlighted that effective digital learning resources in prosthodontic education should promote spatial understanding, encourage active learner engagement, stimulate critical thinking, and connect knowledge to clinical practice. Three-dimensional virtual patients are well suited to address these aspects, offering immersive and interactive scenarios that combine visual realism with clinical relevance [18]. Suarez et al. recently demonstrated the usefulness of artificial intelligence in form of a chatbot [19]. Nevertheless, there are only few studies on virtual patients in dentistry, especially in terms of concept development.

The aim of this study was to develop a concept for the incorporation of virtual patients – defined here as interactive, case-based learning scenarios simulating realistic dentist-patient encounters, including clinical information, patient dialogues, and decision-making tasks - in the dental curriculum of prosthetic dentistry and to evaluate it in terms of a better preparation for real patient contact.

## Materials and methods

### Study design, setting and subjects

During this prospective, controlled trial, the concept was developed and transferred into an e-learning tool at the Department of Prosthodontics at the University Medical Center Hamburg-Eppendorf, Germany. Students’ perceived competences regarding different aspects during comprehensive dental treatments were assessed with self-administered questionnaires before and after applying the e-learning tool. The competence questionnaire at follow-up was supplemented by a questionnaire to evaluate the e-learning tool. The baseline-assessment took place at the beginning of the study year, while the follow-up-assessment and the evaluation were completed when students already had progressed in their studies and in some cases, had their first patient contact. The study lasted around half a year. All surveys were conducted online and anonymously.

Students of third (*n* = 15) and fourth year (*n* = 38) were able to take part in the study voluntarily. These two cohorts differed by only one semester (6th vs. 7th), both belonging to the same stage of study with comparable curricular content and learning objectives; therefore, no substantial difference in prior knowledge was expected. Only participants received access to the e-learning tool. 48 students participated at baseline (response rate: 90%; 14 from third, 34 from fourth year). Students were introduced to the e-learning tool by written instructions, and they were given sufficient time to use it. The follow-up-assessment was completed by 36 students (response rate: 68%) of whom 24 stated to have used the e-learning tool (user group; 5 from third, 19 from fourth year). The non-users (*n* = 12) formed a control group (6 from third, 6 from fourth year), with nine of them commenting they had not had enough time to use the e-learning tool. Illness, lack of activation and exams were each mentioned once, while two participants did not comment any reasons. The responses of the non-users were considered in baseline- and follow-up-assessment of perceived competences, but not in the evaluation of the e-learning tool.

### Development and implementation of e-learning content

**Fig. 1:**
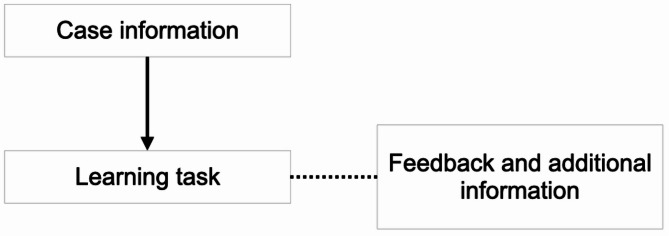
Concept within the learning goals

**Fig. 2:**
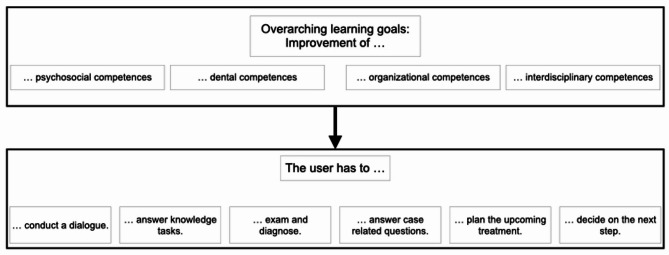
Course structure and composition of the learning goals

The developed concept is based on the procedure of comprehensive dental treatments and has therefore been designed to provide the user with a narrative – from the first appointment including the medical history to the completion of oral rehabilitation. In this context, the e-learning tool emphasized both clinical knowledge and soft skills. Students were required to engage in tasks such as initiating a first conversation with the patient, conducting general and specific anamnesis, and making diagnostic and therapeutic decisions, thereby training both clinical reasoning and communicative competences. These included, for example, performing basic examinations (e.g., charting, periodontal screening, radiographic indications), planning and prioritizing treatment steps across different disciplines (conservative, surgical, and prosthodontic), and practicing patient communication. Rather than simply consuming content, students shall actively participate and interact in form of case-based learning and thus progress within the e-learning environment. In order to avoid discrepancies between examination knowledge and course content in accordance with John Biggs’ Constructive Alignment, overarching learning goals were defined based on the National Competence-Based Learning Objectives Catalogue for Dentistry (NKLZ) as well as the requirements set in the clinical course: psychosocial, interdisciplinary, organizational and dental competences [20, 21]. Depending on which students’ skill shall be promoted, the corresponding module serves as a template for the design of the respective section. The modules consist of three main components: *Case information*, *additional information* and *learning tasks* (Fig. 1). The case information was structured and additional information (e.g., medical and dental knowledge, treatment preparation guidelines, organizational course procedures) was defined to enhance students’ knowledge and prepare them for the course. Each section of the case formed a block for which a learning task was developed according to the chosen learning goals. Answering this task limited the students’ access to the subsequent contents (Fig. 2).

The e-learning tool consisted of three patient cases treated and documented by dentists of the University Medical Center Hamburg-Eppendorf. Those cases presented different clinical situations and involved the prosthetic treatment of (1) a single crown in the maxilla, (2) a single crown in the maxilla and two conventional bridges in the mandible, and (3) a complete denture in the maxilla and a partial denture anchored on double crowns in the mandible. By incorporating cases and exercises of varying levels of difficulty and complexity, the e-learning tool was designed to offer relevant content for students with different levels of prior knowledge, without individual customization. Adapted documentation ensured a variety of topics to be addressed. To design the learning environment as appealing as possible, the available documentation (e.g., dentist’s documentation, x-rays) was used, and patients were visualized by photos. In addition, dialogues between dentist and patient were filmed to present patient contact even more realistic. Those were created in collaboration with the University of Fine Arts, Hamburg, Germany.

The online platform CASUS^®^ (Instruct AG, Munich, Germany) was used to create the cases. It has a linear structure and offers the possibility to implement a wide variety of media and to use different types of tasks. Its use has been tested on multiple devices and browsers to ensure students were independent of specific hardware requirements and can use it anytime and anywhere, assuming they have internet access.

### Assessment of perceived competences

Available questionnaires on student perceptions [16, 22–24] were used as sources for relevant items which were adapted for the purpose of this study. This process resulted in a 14-item questionnaire measuring students’ perceived competence (Appendix 1). The focus was on assessing psychosocial and dental competences. However, interdisciplinary and organizational competences were tested as well. Responses could be made on a 6-point Likert scale ranging from “fully applies” (6) to “not true at all” (1). The questionnaire was complemented by the opportunity for the students at baseline-assessment to express personal expectations by written comments.

### Evaluation of the e-learning tool

An additional questionnaire with 24 items was developed to evaluate the e-learning tool in regard of its functionality and its scope – in its entirety as well as the integrated elements in detail (Appendix 2). Those, evaluated according to their helpfulness, as well as further items referred to applicability and future alignment concluded the evaluation in terms of satisfaction. The content of this questionnaire was developed by selecting and adapting relevant items from studies with comparable assessments [11, 22–27]. Participants responded on a 6-point Likert scale ranging from “fully applies” (6) to “not true at all” (1) and in case of items regarding scope and difficulty on a 5-point Likert scale to allow an undifferentiated positioning. At the end students could express praise, criticism, and suggestions in form of free text comments.

### Statistical analysis

The data of the competence questionnaires and the evaluation questionnaire are presented by item means and standard deviations (SD). In addition, values of the single items were dichotomized – values 4–6 (positive half of the scale) corresponded to agreement, while values 1–3 (negative half of the scale) represented non-agreement. A total mean value of 3.5 determined for each participant was set as the threshold for calculating the overall agreement. When analyzing the scope-specific items, the response “appropriate” was counted as agreement and a distinction between positive and negative disagreement was made. The range of overall agreement was defined as 2.5 to 3.5. To compare perceived competence at baseline and follow-up, changes in scores for each individual item were tested for statistical significance using the Wilcoxon rank-sum test, as data were assessed anonymously and hence pairwise comparisons were not possible. This was supplemented by the calculation of standardized effect sizes [28]. For values of 0.2 the effect was classified as small, for 0.5 as medium, and for 0.8 as large. Analyses were performed separately for users and non-users with the combined baseline data.

Free text comments of the baseline competence questionnaire and the evaluation questionnaire were analyzed based on guidelines on qualitative content analysis [29]. All comments were divided into individual statements, as the students raised several points. For the baseline-assessment they were classified according to their superior topic, while for the evaluation questionnaire at follow-up they were additionally divided into praise, criticism, and suggestions.

All analyses were performed using statistical software package Stata (Stata Statistical Software: Release 17. Stata Corp LP, College Station, TX, US), with the probability of a type I error set at the 0.05 level.

## Results

### Characterization of participants

At baseline, the mean age of all 48 participants was 23.3 (SD: 2.4) years and almost one-third were female (Table 1). Most participants were fourth-year students and did not have dental or medical prior knowledge.

**Table 1:** Characteristics of participants at baseline- and follow-up-assessment

	Baseline	Follow-Up	
	All	Users	Non-users
	*n* = 48	*n* = 24	*n* = 12
	Mean (SD) or n (%)		
Age			
Years	23.3 (2.4)	23.3 (1.9)	24.7 (3.5)
Gender			
Male	17 (35.4)	6 (25.0)	8 (66.7)
Female	31 (64.6)	18 (75.0)	4 (33.3)
Study year			
Third	14 (29.2)	5 (20.8)	6 (50.0)
Fourth	34 (70.8)	19 (79.2)	6 (50.0)
Prior knowledge			
Yes	12 (25.0)	7 (29.2)	4 (33.3)
No	36 (75.0)	17 (70.8)	8 (66.7)

### Perceived competences at baseline

For the individual items of the competence questionnaire, means for all participants ranged from 2.0 for *dental emergencies* to 3.1 for *prosthetic treatment procedures*, resulting in a total mean score of 2.5. The overall agreement was 8.3% (Table 2).

**Table 2:** Item scores of competence questionnaire at baseline and follow-up, and change between both assessments for users and non-users

		Baseline		Follow-Up				Change			
				Users		Non-users		Users		Non-users	
		*n* = 48		*n* = 24		*n* = 12		*n* = 24		*n* = 12	
Domain	Item	Mean (SD)	%	Mean (SD)	%	Mean (SD)	%	p-value	Effect Size	p-value	Effect Size
Psychosocial competences	(First) patient contact	2.6 (1.1)	18.8	4.3 (1.0)	79.2	3.3 (1.2)	33.3	< 0.001	1.6	0.116	0.6
	Communication	2.7 (1.4)	27.1	4.4 (1.0)	87.5	3.3 (1.2)	41.7	< 0.001	1.3	0.126	0.4
	Handling	2.6 (1.2)	20.8	4.4 (0.8)	87.5	3.4 (1.0)	41.7	< 0.001	1.6	0.025	0.7
	Anamnestic interview	2.6 (1.4)	27.1	4.4 (1.0)	87.5	3.3 (1.2)	50.0	< 0.001	1.4	0.089	0.5
Interdisciplinary competences	Health impaired patients	2.4 (1.3)	16.7	3.7 (1.1)	54.2	2.7 (1.1)	25.0	< 0.001	1.0	0.448	0.2
	Medication, side effects and measures	2.2 (1.1)	10.4	3.4 (1.2)	45.8	2.8 (1.1)	33.3	< 0.001	1.0	0.124	0.5
Organizational competences	Organizational processes	2.3 (1.2)	10.4	3.5 (1.3)	50.0	2.8 (1.1)	33.3	< 0.001	1.0	0.190	0.4
	Planning presentation	2.3 (1.2)	10.4	3.0 (1.3)	33.3	2.8 (1.1)	25.0	0.023	0.6	0.295	0.4
Dental competences	Treatment planning	2.7 (1.3)	25.0	4.0 (1.1)	66.7	3.6 (1.1)	58.3	< 0.001	1.0	0.024	0.7
	Therapy options	2.7 (1.3)	25.0	3.5 (1.0)	58.3	3.0 (1.0)	33.3	0.002	0.7	0.341	0.2
	Decision making	2.5 (1.2)	18.8	3.4 (1.1)	50.0	3.0 (1.0)	41.7	0.002	0.8	0.168	0.4
	Prosthetic planning	2.8 (1.1)	20.8	3.3 (1.2)	37.5	2.8 (1.2)	41.7	0.096	0.4	0.745	0.1
	Prosthetic treatment procedures	3.1 (1.2)	37.5	3.6 (1.0)	66.7	3.1 (0.8)	33.3	0.038	0.4	0.859	−0.04
	Dental emergencies	2.0 (1.1)	6.3	2.9 (1.3)	20.8	2.5 (1.0)	16.7	0.002	0.8	0.077	0.5
Total		2.5 (1.2)	8.3	3.7 (1.2)	75.0	3.0 (1.1)	33.3				

Total agreement: proportion of participants whose total mean score was 3.5 or above

The qualitative analysis of 25 students’ comments resulted in 36 isolated statements. 28 of them expressed the wish to be better prepared. Twelve of these referred to being better prepared in general, 16 statements were specific to treatment planning and procedures (*n* = 8), first patient contact (*n* = 6), or organizational processes (*n* = 2). Furthermore, feeling more confident was mentioned five times, and competence regarding diseases and medication was mentioned once.

### Perceived competences at follow-up

In the group of users, the items *communication*, *handling* and *anamnestic interview* reached the highest mean score (4.4) representing highest perceived competences, whereas the item *dental emergencies* was rated lowest with 2.9 (Table 2). All items in total had a mean score of 3.7 and the overall agreement reached a value of 75.0%. For non-users, all items had lower mean scores than for users, with the item *treatment planning* scoring highest (3.6) and the item *dental emergencies* scoring lowest (2.5). Overall, the mean score was 3.0 and the agreement was 33.3% for all items combined.

### Comparison of perceived competence between baseline and follow-up

From baseline to follow-up, the overall agreement in users increased from 8.3% to 75.0% and a statistically significant improvement in almost every item was observed after applying the e-learning tool (Table 2). Only *prosthetic planning* (*p* = 0.096) showed no statistically significant improvement. The effect size ranged from 1.6 to 0.4 in this group.

The results differed when compared to the students who did not use the e-learning tool. The overall agreement at follow-up was only 33.3%. Statistically significant improvements were only found for the items *handling* (*p* = 0.025) and *treatment planning* (*p* = 0.024). These observations were confirmed by the effect sizes which showed smaller values compared to the users, with values ranging from − 0.04 to 0.7 for non-user.

### Evaluation of the e-learning tool

The results of the items that were answered on a 6-point Likert scale are shown in Table 3. Highest mean was 5.3 with an agreement of 95,7% for the items *expansion* and *recommendation*. Although technical problems did not occur often, *technical support* reached the lowest mean with 3.9 and an agreement of 71.4%. The total mean score was 4.9 and the overall agreement 75.0%. Table 4 shows the results of the items that were answered on a 5-point Likert scale. The overall agreement was 87.5% with a positive disagreement of 12.5%.

**Table 3:** Item scores for the evaluation of the functionality and the satisfaction

		Follow-Up	
		Users	
		*n* = 24	
Domain	Item	Mean (SD)	Agreement (%)
Functionality	Users interface design	5.1 (0.9)	91.7
	Users interface handling	5.2 (0.9)	91.7
	Technical problems	5.0 (1.6)	79.2
	Technical support	3.9 (1.8)	71.4
Satisfaction	Exercises understandability	4.8 (0.8)	95.7
	Patient cases helpfulness	5.2 (1.0)	95.7
	Patient videos helpfulness	4.7 (1.2)	87.0
	Exercises helpfulness	4.9 (0.9)	95.7
	Answer comments helpfulness	5.0 (0.9)	95.7
	Competences improvement	4.6 (1.1)	87.0
	Motivation	4.3 (1.2)	78.3
	Enjoyment	4.5 (1.3)	82.6
	Expansion	5.3 (0.9)	95.7
	Recommendation	5.3 (1.2)	95.7
Total		4.9 (1.1)	91.7

Total agreement: proportion of participants whose total mean score was 3.5 or above

**Table 4:** Item scores for the evaluation of the scope

		Follow-Up			
		Users			
		*n* = 24			
Domain	Item	Mean (SD)	Negative disagreement (%)	Agreement (%)	Positive disagreement (%)
Scope	Quantity patient videos	2.6 (0.6)	0.0	60.9	39.1
	Quantity exercises	2.7 (0.6)	0.0	73.9	26.1
	Quantity possible answers	2.7 (0.6)	0.0	78.3	21.7
	Quantity answer comments	3.0 (0.5)	13.0	78.3	8.7
	Processing time	2.4 (0.7)	0.0	52.2	47.8
	Patient videos length	2.8 (0.5)	0.0	82.6	17.4
	Exercises length	2.8 (0.5)	0.0	87.0	13.0
	Answer comments detailedness	3.2 (0.8)	25.0	66.7	8.3
	Exercises difficulty	2.8 (0.7)	8.7	69.6	21.7
Total		2.8 (0.6)	0.0	87.5	12.5

Negative disagreement: (rather) too low, short, tight, easy/agreement: appropriate/positive disagreement: (rather) too high, long, detailed, difficult total agreement: proportion of participants whose total mean score was between 2.5 and 3.5

Free text comments were provided by six participants, resulting in ten separate statements. Four were praise, four were criticism, and the rest were suggestions. The idea was praised, and the e-learning module was described as “very helpful” (*n* = 2) and “appealingly designed”. Criticisms included technical problems, “lack of basic knowledge to work through the questions,” and limitations of cloze texts (e.g., synonyms). Recommendations included “more cases,” more detailed answer comments, and correction of minor errors.

## Discussion

Findings of this study indicate that students did not feel well prepared for the clinical course. A better preparation of both, participants as well as students who did not use the e-learning module, was observed in the follow-up-assessment. However, a statistically significant improvement was almost always observed among users, while this was rarely the case in non-users. The final evaluation demonstrated a high level of user’s satisfaction, who would recommend it to fellow students and advocated an expansion.

The observations at baseline were comprehensible, as standardized cases on phantom patients mainly prepare for prosthetic treatment procedures and do not feature any complexity. Neither do general diseases occur, nor medications need to be considered. Furthermore, phantom patients do not provide any feedback, resulting in a lack of training in communication and appropriate handling. The significant improvement after the participation in the e-learning was consistent with the realization of students’ previously set expectations. This was most evident in their perception of improved communication skills and the emphasis on visualization and communication to make the experience as realistic as possible. The improvement observed among non-users can be explained by the progress they have made in their studies. In particular, the comparison of those with the participants revealed the e-learning-tool as the source of improvement. The positive final evaluation underlines the added value and the perceived improvement.

Even though there are only a few studies on the integration of virtual patients into the dental curriculum, their results confirm the benefits and the acceptance by students observed here [30–33]. There are differences in the topics addressed and the number of virtual patients as well as the timing and duration of the assignment vary. Courses were evaluated differentially with respect to the methodical approach. In Malmö, anamnestic interviews conducted by students with their first real patient were recorded [34]. While the test group was allowed to use the virtual patient beforehand, the control group had the opportunity to do so afterwards. A more comprehensive anamnestic interview turned out and empathy at a higher level was observed. This is consistent with the results of our study that students felt better prepared, especially for their first patient contact and for conducting an anamnestic interview. A new virtual learning experience was piloted and evaluated in dental, medical and pharmaceutical courses at the Karolinska Institute and the Uppsala University [35]. As the students in our study, students, and teachers, regard the virtual patient cases as a valuable supplement to the curriculum.

Greatest strength of the concept is its clear structure, which not only simplifies the use, but also provides developers with a guidance for case creation. No matter whether they are teachers or students. Application by the latter involved them in process actively and presented knowledge to their fellow students from a similar perspective. As a result, the process as well as the generated e-learning module were even more student-centered. However, teachers were indispensable as supervision of created content in terms of its accuracy was necessary. Assuming students’ access to required hardware and internet, a further strength arises, as it can be used anytime and anywhere, thus providing the opportunity for flexible learning. No notable problems regarding access or usability of the e-learning tool were reported in the present study. Nevertheless, it must be considered that in different settings, variations may occur if students are less familiar with technology or have unequal access to hardware and internet, which could limit their ability to use such tools effectively. Another strength results from the combined quantitative and qualitative evaluation, providing a deeper insight in participant’s satisfaction level as well as allowing the optimization of the e-learning tool. As the e-learning tool was voluntary and not all of the participants used it, a control group was formed, enabling a comparison not only between before and after the use, but also a comparison between two groups towards the end of the study. The study has its limitations, too. It was conducted only in one setting. But since students and curricula at other study sites are similar, an achievement of related results can be assumed here as well. Another limitation is that the present concept was developed and evaluated exclusively in the context of prosthodontic cases. However, since students are required to make interdisciplinary decisions as part of the synoptic treatment concept, the approach is in principle transferable to other dental disciplines, where comparable outcomes may also be expected. It should be mentioned that the questionnaires were not tested in advance, but comparability is ensured as they are based on similar studies. In the present study, a 6-point Likert scale was chosen to reduce the tendency of respondents to select a neutral midpoint and thereby to obtain clearer directional responses. While this approach has the disadvantage of not allowing participants to express a strictly neutral position, it was considered appropriate to minimize central tendency bias and provided a more differentiated picture of students’ evaluations, despite the limitation of not capturing neutrality explicitly. As both assessments were conducted anonymously, the samples had to be viewed and analyzed independently, which reduced the statistical power. Nevertheless, the p-values were very small and therefore statistically significant. Another limitation was the number of participants, which resulted from small courses in general and probably as a result of high workload due to courses, lectures, and seminars. It must be considered that students who voluntarily used the e-learning tool were likely to be more motivated and generally higher-performing than those who did not, which may have influenced the reliability of their responses. In contrast, less motivated students may have been more likely to abstain from using the tool and to provide less differentiated answers. Nevertheless, the overall positive development observed in students’ self-assessments was consistent with our expectations and supports the plausibility of the findings. The lack of objective assessments in regard of clinical knowledge retention and growth limits a statement on whether a better students’ performance can be expected. Another potential limitation is that participants came from two consecutive semesters (6th and 7th), which introduces a certain degree of heterogeneity. However, as both semesters belong to the same stage of the curriculum with comparable content and learning objectives, no substantial bias in prior knowledge is expected. Future studies may consider stratified randomization or separate analyses by semester to further minimize this potential source of variation. However, these limitations do not affect the significance of the results.

The e-learning tool provided students with their first opportunity to engage with virtual patients and may address their growing demand for digital learning formats [36]. As a permanent incorporation into the existing curriculum, they can apply and deepen their knowledge, gain new one and train for the work with real patients by improving their competencies. The optimized learning experience may result in a better preparation for their professional life and therefore better dentists. Independent use as well as the collaboration with other students have the potential to intensify the learning experience. By doing so, they can benefit from experiences of higher semesters, while those repeat their knowledge. In addition, virtual patients can be used as objective instruments in examinations. Realization of the desired expansion and the concept serving as a guideline for other universities may point the way to joint use among universities. The more cases available, the more learning goals of different dental disciplines can be addressed. This leads to a more realistic presentation out of standardized boundaries - from a dental, an interdisciplinary, and even a social perspective.

## Conclusion

The integration of virtual patients into preclinical prosthodontics education proved feasible, well-received, and effective in enhancing students’ perceived competence. The concept supports a more realistic, self-directed, and patient-centered learning experience. Virtual patients can bridge the gap between preclinical training and clinical practice and should be further developed and, to provide a more comprehensive evaluation of competence development, evaluated using objective outcome measures in addition.

## Supplementary Information

Supplementary Material 1.

Supplementary Material 2.

## Data Availability

The datasets generated and analyzed during the current study are available from the corresponding author on reasonable request.

## Abbreviations

VP: Virtual Patients
NKLZ: National Competence-Based Learning Objectives Catalogue for Dentistry
SD: Standard Deviation
