# Supplementary material for: Bridging the gap to clinical practice: a concept for virtual patients in preclinical education in prosthetic dentistry
Source: BMC Med Educ. 2025 Oct 16;25:1429. doi: 10.1186/s12909-025-08097-4 (PMC12529795; doi:10.1186/s12909-025-08097-4)
Supplement: Supplementary file 1 — Supplementary Material 1. [file 12909_2025_8097_MOESM1_ESM.docx]

| No. | Item | |
| --- | --- | --- |
|  | German | English |
| 1 | Ich fühle mich gut auf den ersten Patientenkontakt vorbereitet. | I feel well prepared for the first patient contact. |
| 2 | Ich fühle mich gut auf die Kommunikation mit Patienten vorbereitet. | I feel well prepared to communicate with patients. |
| 3 | Ich fühle mich gut auf den Umgang mit Patienten vorbereitet. | I feel well prepared to handle with patients. |
| 4 | Ich fühle mich gut auf das Führen eines Anamnesegesprächs vorbereitet. | I feel well prepared to conduct an anamnestic interview. |
| 5 | Ich fühle mich gut auf die Zusammenarbeit mit gesundheitlich beeinträchtigten Patienten vorbereitet. | I feel well prepared to work with health-impaired patients. |
| 6 | Ich fühle mich gut auf Medikamente, deren Nebenwirkungen und zu beachtende Maßnahmen vorbereitet. | I feel well prepared for medication, its side effects and measures to be taken. |
| 7 | Ich fühle mich gut auf die organisatorischen Abläufe im klinischen Behandlungskurs. | I feel well prepared for the organisational processes in the clinical treatment course. |
| 8 | Ich fühle mich gut auf die Behandlungsplanung gemäß des Synoptischen Behandlungskonzepts vorbereitet. | I feel well prepared for treatment planning according to the Synoptic Treatment Concept. |
| 9 | Ich fühle mich gut auf die unterschiedlichen Therapieoptionen vorbereitet. | I feel well prepared for the different therapy options. |
| 10 | Ich fühle mich gut auf die Entscheidungsfindung bei der Therapie vorbereitet. | I feel well prepared for decision making in therapy. |
| 11 | Ich fühle mich gut auf die prothetische Planung vorbereitet. | I feel well prepared for the prosthetic planning. |
| 12 | Ich fühle mich gut auf die Planungsvorstellung vorbereitet. | I feel well prepared for the planning presentation. |
| 13 | Ich fühle mich gut auf die prothetischen Behandlungsabläufe vorbereitet. | I feel well prepared for the prosthetic treatment procedures. |
| 14 | Ich fühle mich gut auf die Konfrontation mit zahnmedizinischen Notfällen vorbereitet. | I feel well prepared to confront with dental emergencies. |
| Response scale: German: trifft überhaupt nicht zu - trifft weitgehend nicht zu - trifft eher nicht zu - trifft eher zu - trifft weitgehend zu - trifft völlig zu English: not true at all - largely not true - rather not true - rather applies - largely applies - fully applies | | |
