# Supplementary material for: Bridging the gap to clinical practice: a concept for virtual patients in preclinical education in prosthetic dentistry
Source: BMC Med Educ. 2025 Oct 16;25:1429. doi: 10.1186/s12909-025-08097-4 (PMC12529795; doi:10.1186/s12909-025-08097-4)
Supplement: Supplementary file 2 — Supplementary Material 2. [file 12909_2025_8097_MOESM2_ESM.docx]

| No. | Item | |
| --- | --- | --- |
|  | German | English |
| 1 | Die Benutzeroberfläche ist übersichtlich gestaltet.^1^ | The users interface is clearly designed.^1^ |
| 2 | Die Benutzeroberfläche ist einfach zu handhaben.^1^ | The users interface is easy to handle.^1^ |
| 3 | Es sind keine technischen Probleme aufgetreten.^1^ | No technical problems have occurred.^1^ |
| 4 | Bei technischen Problemen wurde ich unterstützt. (Nur zu beantworten, wenn technische Probleme aufgetreten sind.)^1^ | I was supported in case of technical problems. (To be answered only if technical problems occurred).^1^ |
| 5 | Ich habe mir die Patientenvideos angesehen.^1^ | I watched the patient videos.^1^ |
| 6 | Die Anzahl der Patientenvideos ist … .^2^ | The quantity of patient videos is … .^2^ |
| 7 | Die Anzahl der Aufgaben ist … .^2^ | The quantity of exercises is … . ^2^ |
| 8 | Die Anzahl der Antwortmöglichkeiten ist … .^2^ | The quantity of possible answers is … .^2^ |
| 9 | Die Anzahl der Antwortkommentare ist … .^2^ | The quantity of answer comments is … .^2^ |
| 10 | Die Bearbeitungszeit der Fälle ist … .^3^ | The processing time of the cases is … .^3^ |
| 11 | Die Patientenvideos sind … .^3^ | The patient videos are … .^3^ |
| 12 | Die Aufgabenstellungen sind … .^3^ | The exercises are … .^3^ |
| 13 | Die Antwortkommentare sind … .^4^ | The answer comments are … .^4^ |
| 14 | Die Aufgabenstellungen sind verständlich.^1^ | The exercises are understandable.^1^ |
| 15 | Die Aufgaben sind … .^5^ | The exercises are … .^5^ |
| 16 | Die Patientenfälle sind hilfreich.^1^ | The patient cases are helpful.^1^ |
| 17 | Die Patientenvideos sind hilfreich.^1^ | The patient videos are helpful.^1^ |
| 18 | Die Aufgaben sind hilfreich.^1^ | The exercises are helpful.^1^ |
| 19 | Die Antwortkommentare sind hilfreich.^1^ | The answer comments are helpful.^1^ |
| 20 | Das Arbeiten mit dem Lernmodul hat mir gezeigt in welchen Kompetenzen ich mich verbessern kann.^1^ | Working with the learning module has shown me in which competences I can improve.^1^ |
| 21 | Das Arbeiten mit dem Lernmodul hat mich motiviert mich weiter mit den Inhalten zu beschäftigen.^1^ | Working with the learning module has motivated me to further engage with the content.^1^ |
| 22 | Das Arbeiten mit dem Lernmodul hat mir Spaß gemacht.^1^ | I enjoyed working with the learning module.^1^ |
| 23 | Das Lernmodul sollte ausgebaut werden.^1^ | The learning module should be expanded.^1^ |
| 24 | Ich würde das Lernmodul nachfolgenden Semestern empfehlen.^1^ | I would recommend the learning module to subsequent semesters.^1^ |
| Response scale: German: 1: trifft überhaupt nicht zu - trifft weitgehend nicht zu - trifft eher nicht zu - trifft eher zu - trifft weitgehend zu - trifft völlig zu 2: zu hoch - eher zu hoch - angemessen - eher zu niedrig - zu niedrig 3: zu lang - eher zu lang - angemessen - eher zu kurz - zu kurz 4: zu ausführlich - eher zu ausführlich - eher zu knapp - zu knapp 5: zu schwierig - eher zu schwierig - angemessen - eher zu leicht - zu leicht English: 1: not true at all - largely not true - rather not true - rather applies - largely applies - fully applies 2: too high - rather too high - appropriate - rather too low - too low 3: too long - rather too long - appropriate - rather too short - too short 4: too detailed - rather too detailed - appropriate - rather too tight - too tight 5: too difficult - rather too difficult - appropriate - rather too easy - too easy | | |
